# Predictive models of medication non-adherence risks of patients with T2D based on multiple machine learning algorithms

**DOI:** 10.1136/bmjdrc-2019-001055

**Published:** 2020-03-09

**Authors:** Xing-Wei Wu, Heng-Bo Yang, Rong Yuan, En-Wu Long, Rong-Sheng Tong

**Affiliations:** 1 Personalized Drug Therapy Key Laboratory of Sichuan Province, School of Medicine, University of Electronic Science and Technology of China, Chengdu, China; 2 Department of Pharmacy, Sichuan Academy of Medical Sciences and Sichuan Provincial People’s Hospital, Chengdu, China; 3 School of Pharmacy, Chengdu Medical College, Chengdu, China; 4 Endocrine Department, Sichuan Academy of Medical Sciences and Sichuan Provincial People’s Hospital, Chengdu, China

**Keywords:** adherence, type 2 diabetes, prediction and prevention, personality

## Abstract

**Objective:**

Medication adherence plays a key role in type 2 diabetes (T2D) care. Identifying patients with high risks of non-compliance helps individualized management, especially for China, where medical resources are relatively insufficient. However, models with good predictive capabilities have not been studied. This study aims to assess multiple machine learning algorithms and screen out a model that can be used to predict patients’ non-adherence risks.

**Methods:**

A real-world registration study was conducted at Sichuan Provincial People’s Hospital from 1 April 2018 to 30 March 2019. Data of patients with T2D on demographics, disease and treatment, diet and exercise, mental status, and treatment adherence were obtained by face-to-face questionnaires. The medication possession ratio was used to evaluate patients’ medication adherence status. Fourteen machine learning algorithms were applied for modeling, including Bayesian network, Neural Net, support vector machine, and so on, and balanced sampling, data imputation, binning, and methods of feature selection were evaluated by the area under the receiver operating characteristic curve (AUC). We use two-way cross-validation to ensure the accuracy of model evaluation, and we performed a posteriori test on the sample size based on the trend of AUC as the sample size increase.

**Results:**

A total of 401 patients out of 630 candidates were investigated, of which 85 were evaluated as poor adherence (21.20%). A total of 16 variables were selected as potential variables for modeling, and 300 models were built based on 30 machine learning algorithms. Among these algorithms, the AUC of the best capable one was 0.866±0.082. Imputing, oversampling and larger sample size will help improve predictive ability.

**Conclusions:**

An accurate and sensitive adherence prediction model based on real-world registration data was established after evaluating data filling, balanced sampling, and so on, which may provide a technical tool for individualized diabetes care.

**Significance of this study**

**What is already known about this subject?**

- Medication adherence is a key to diabetes care.
- Proper care can help improve medication adherence.

**What are the new findings?**

- The newly developed model has a good performance to predict the risk of non-medication adherence in diabetics.

**How might these results change the focus of research or clinical practice?**

- This model can be used to filter patients with high risk of non-medication adherence and precise educational interventions can be conducted.

## Introduction

Type 2 diabetes (T2D), one of the most popular disorders all over the world,^1^ is a chronic condition that requires long-term treatment to improve life quality and reduce the probability of related disability and death.^2–4^ Studies have confirmed that good medication adherence is important for patients with T2D to improve glycemic control, avoid complications, and reduce overall health expenditure.^5 6^ Interventions such as integrative health coaching can significantly improve medication adherence.^7–9^ Considering the large crowd and complex features of patients with T2D, identifying high-risk groups with poor medication adherence and carrying out precise educational interventions are feasible measures to improve the overall disease control.

Studies found that numerous factors may be associated with medication adherence in patients with T2D, including the duration of the disease, mental state, anxiety, depression, irritability, smoking, cost, and so on.^5 10 11^ These factors vary with study designs and regions. That may be resulted from the differences in the selection of variables, patients’ income levels, education levels, cultural characteristics, living habits, and so on. Therefore, it is necessary to explore the main influencing factors of local patients’ medication compliance and to establish a model that can accurately predict therapeutic compliance in specific regions.^12 13^ Despite several compliance-related predictive models that have been reported recently in patients with tuberculosis^14^ and heart failure,^15^ studies on patients with T2D based on multivariate machine learning algorithms have not been retrieved.

In this study, a rigorous and comprehensive questionnaire survey was conducted and dozens of machine learning-based models were built and assessed. In addition, we also examined the impacts of data preprocessing, modeling methods and different sample sizes on prediction performance.

## Research design and methods

### Study design, study area, and participants

A survey was conducted in the outpatient clinic of Sichuan Provincial People’s Hospital from April 2018 to March 2019. The hospital mainly serves patients from Sichuan Province, a populous province in southwestern China. A total of 630 patients were approached, and 401 completed the survey. Researchers conducted a face-to-face questionnaire survey and filled out questionnaires according to the answers from the patients who participated in the survey.

Patients with T2D were selected according to the criteria: (1) examined HbA1c on the day of the questionnaire, and (2) willing to take part in the survey and to provide information to the investigators. Patients were eliminated if he or she (1) was a non-T2D patient, (2) did not receive hypoglycemic agency treatment, (3) less than 18 years old, and (4) with a short life expectancy.

### Data collection

The data in this study were collected from electronic medical records (EMR) and face-to-face questionnaires. Clinical laboratory results were collected according to EMRs. The results of the previous examination and the duration from the last test were recalled by patients and, if necessary, confirmed by telephone after they returned home.

### Questionnaires

The questionnaire consists of five parts. The first part is about basic characteristics, including age, gender, occupation, family history, and so on. The second part is involved in the information related to diabetes, including self-glycemic monitoring, diabetes course, medication regimen, duration of medication regimen, the test results of HbA1c and blood glucose at the time of the questionnaire. The third part is referred to the other clinical information, including the use of Chinese traditional medicine products, surgical histories, comorbidities, and concomitant medications. The fourth part is about exercise, diet, and mental state. The last part is information related to adherence, in which we recorded how many medications should be taken, how many were prescribed and how many were left. The adherence status, the medication possession ratio (the proportion of medication’s available days, higher than 80% is regarded as good medication compliance^16 17^), was determined as the target variable.

### Data blindness

All variable names were encoded to X1–X44 to achieve statistical blindness in this study. Data analysis was performed using the encoded variable names. The variables were unblinded after the model evaluation processes.

### Data cleaning

Variables with more than 50% missing or a certain category’s proportion greater than 80% were excluded. The maximum likelihood ratio method was used to assess the correlations between the input variables and the target variable. Variables with p value >0.1 were considered unimportant and were excluded after the likelihood ratio test. Data filling was performed using mean value (for numerical variables), median (for ordinal variables) or mode (for nominal variables).^18^ Outlier values were modified as the maximum or minimum of normalized values.

Because of the proportional imbalance between good and non-adherence patients, sampling methods, including oversampling (four times for patients with poor compliance) and undersampling (50% for patients with good adherence), were taken to make up the shortage caused by the imbalanced sample size between the different levels of the target variable. Unbalanced data were analyzed simultaneously to evaluate the risk of overfitting on account of balancing dispose.

### Data partition

There were two data partitioning processes in this study for two-way cross-validation. The first was conducted before data cleaning. In this process, the original data were randomly divided into two subsets (named set 1 and set 2) in a ratio of 8:2, which would be used for model establishment and verification, respectively. During the second partition, set 1 was divided into a training set and a testing set by 7:3 after data cleaning. The training set would be used to build machine learning models, and the testing set would be used to evaluate the fitting effectiveness of the models.

### Variable selection

The forward, backward and stepwise methods of logistic regression were used to screen significant variables. The continuous variables were grouped (or not) according to the IQR, in which process was named binning, aimed to facilitate interpretation.

### Model validation

The predictive performances of the models were evaluated by the area under the receiver operating characteristic curve (AUC). AUC of every data set (training set of set 1, testing set of set 1, and set 2) was calculated to examine the sensitivity and specificity of models. Due to oversampling dispose, a number of duplicate records would be generated, which means some records might be used both to build the models and to validate the models, and cause the risk of overly optimistic estimation of the models’ prediction effects. So, AUC of set 2, which was partitioned before oversampling, would be used as the best model evaluation indicator. The overfitting risks of the models were assessed by OF1, which was calculated using the formula: AUC_Set 1 training set_/AUC_Set 1 testing set_, and OF2, AUC_Set 1 testing set_/AUC_Set 2_. Higher levels of OF1 and OF2 indicate more serious overfitting risks. Tenfold independent repeated values of the above indexes were generated by changing the random seed number in the first data partition process 10 times.

### Model building

More than a dozen classification algorithms were applied and assessed in this study. The proposed models included C 5.0 model (marked as $C), logistic regression model (marked as $L), decision list, Bayesian network (marked as $B), discriminant model (marked as $D), KNN algorithm (marked as $KNN), LSVM, random forest, SVM (marked as $S), Tree-AS, CHAID (marked as $R), Quest, C&R Tree (marked as $R), Neural Net (marked as $N), and the ensemble model (marked as $XF). The ensemble models would summarize the output of the best five models (assessed by AUC) and generate their outputs according to the voting principle.

### Sample size assessment

The model with the largest AUC was selected, and 10 subsets, 10%–100% in a step of 10% of the total sample size randomly extracted, were used to build models in order to evaluate the influence of different sample sizes on the predictive ability. Every subset was divided into a training set and a testing set by 7:3 and the AUC calculated from the testing set was used for sample size examination. Ten independent replicate results were generated for each model by transforming random sampling seeds.

### Statistical description and hypothesis testing

Continuous variables were expressed as mean±SD and counting variables were expressed in terms of frequency. Student’s t-test and signed-rank tests were used to test the difference between paired quantitative data. T-test and general linear models for analysis of variance were used as parameter test approaches. Non-parametric tests were implemented in Wilcoxon rank-sum analysis and Kruskal-Wallis test. If the data were normally distributed and the variances were equal, an appropriate parameter test was used, otherwise, a non-parametric test was used to realize hypothesis testing. Spearman correlation analysis was used as correlation analysis approach between two continuous variables. Multiple linear regression was used for multivariate analysis, and during which process variance inflation factor and standardized estimate (SE) were used to assess the multicollinearity risk and the weight of the affecting to the dependent variable.

Excel 2016 was used to summarize the data. Data cleaning and modeling were completed using IBM SPSS Modeler V.18.0 software. Variable screening, hypothesis testing, and regression analysis were performed using SAS V.9.21 (SAS Institute). The figure in the sample size verification section was plotted using Prism for Windows 6 software (GraphPad Software).

## Results

### Respondent population

A total of 401 patients out of 630 candidates completed the survey, among which 244 were male and 157 were female. The mean age was 58.9±12.2 years. Eighty-five patients were defined as poor medication adherence (21.20%). For detailed patients’ characteristics see table 1. The rate of data missing was 10.67% and the main reasons were the missing of patients’ memory or that the respondents were not willing to provide certain information.

**Table 1:** Demographic and clinical data of participants

	Parameter	Value (n=401)
Age (years)	n	401
	Mean±SD	58.9±11.89
	Median	58
	Minimum, maximum	27, 85
Gender	n	401
	Male	244 (60.8%)
	Female	157 (39.1%)
Weight (kg)	n	397
	Mean±SD	65.0±10.28
	Median	65
	Minimum, maximum	42, 110
Marital status	n	396
	Married/living as married/civil partnership	393 (99.2%)
	Single/never married	2 (0.4%)
	Divorced or separated	2 (0.4%)
Employment status	n	399
	Unemployed	58 (14.5%)
	Employed	149 (37.3%)
	Retirement	191 (47.9%)
	Other	1 (0.2%)
Highest level of education	n	399
	Illiteracy	41 (10.3%)
	Junior middle school	128 (32.1%)
	High school or special secondary school	130 (32.6%)
	College and above	100 (25.1%)
Family history of diabetes	n	391
	Yes	122 (31.2%)
	No	269 (68.8%)
Previous HbA1c value	n	264
	<7%	97 (36.7%)
	7%–9%	126 (47.7%)
	>9%	41 (15.5%)
The interval between the last HbA1c measurement and the present (days)	n	267
	Mean±SD	227.9±271.52
	Median	150
	Minimum, maximum	2, 2920
The course of diabetes (months)	n	401
	Mean±SD	89.7±76.44
	Median	72
	Minimum, maximum	1, 480
Regular monitoring of fasting blood glucose frequency	n	401
	Irregular monitoring	71 (17.7%)
	Two or three times a week	156 (38.9%)
	Three or four times a month	129 (32.1%)
	Two or three times in 3 months	45 (11.2%)
Fasting blood glucose value (mmol/L)	n	325
	3.8–6.1	23 (7.1%)
	6.1–7	96 (29.5%)
	≥7	206 (63.3%)
Complications	n	401
	Yes	42 (10.5%)
	No	359 (89.5%)
Exercise intensity	n	401
	No exercise	44 (10.9%)
	Low-intensity exercise (eg, walking)	269 (67.0%)
	Medium-intensity exercise (eg, fast walking, jogging)	57 (14.2%)
	High-intensity exercise (eg, fitness, cycling, dancing)	31 (7.7%)
Exercise time (min)	n	401
	Mean±SD	63.3±71.66
	Median	60
	Minimum, maximum	0, 600
Eat reasonably	n	401
	Yes	294 (73.3%)
	No	107 (26.7%)
Sleep status	n	401
	Good	214 (53.3%)
	Ordinary	120 (29.9%)
	Lose sleep	67 (16.7%)
Psychological status	n	401
	Optimistic	247 (61.6%)
	Ordinary	144 (35.9%)
	Depressed	10 (2.5%)
Compliance	n	401
	Good	316 (78.8%)
	Poor	85 (21.2%)

n, number of respondents.

### Data stream

**Figure 1:**
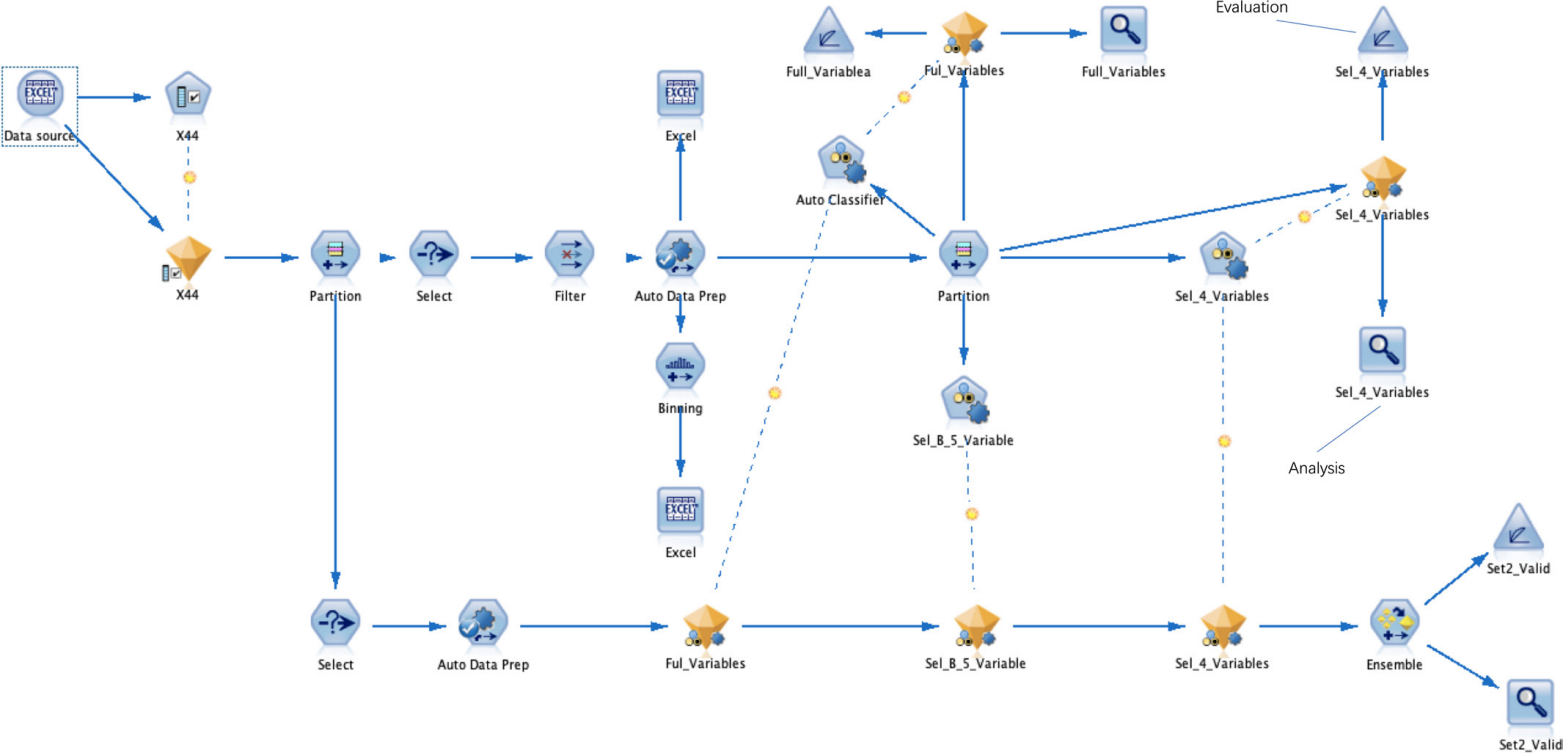
The data flowed into the ‘Partition’ node after feature selection. The ‘Auto Data Prep’ node was used for data filling, the ‘Balance’ node performed a data balanced sampling process, and the ‘Binning’ node was applied for data binning. The ‘Partition’ node divided set 1 into a training set and a testing set, used the ‘Auto Classifier’ node to build various classification models, and used the ‘Analysis’ and ‘Evaluation’ nodes to output the AUC values and curve figure of each model. Use the ‘Select’ node to select the ‘Set 2’ data set. The set 2 set was concatenated with the models established above and the ensemble model of them, and the AUC values and graphs of all models were output using the ‘Analysis’ and ‘Evaluation’ nodes. AUC, area under the receiver operating characteristic curve.

A process framework of the data flow is shown in figure 1. Data flow through each node according to a predetermined schedule.

### Feature selection

Three variables were excluded due to too many missing values. Four variables were excluded because the proportion of certain categories was too large, and 18 variables were excluded because of the low correlation with the target variable. Thereby, a total of 16 variables were used for modeling, including the last HbA1c value, fasting glucose, age, diet adjustment or not, weight, cost of hypoglycemic drugs, duration of current treatment regimen, body mass index (BMI), working status, the duration since the prior blood glucose test, dyslipidemia, and so on.

### Model establishment and evaluation

In this study, 300 machine learning models were developed based on 30 algorithms varying from whether imputing or not, sampling methods (unbalanced, oversampling and undersampling) and variable screening methods (forward, backward and stepwise). By changing the seed value when data partition, each modeling algorithm would be built and tested using 10 separate training and testing data sets, and generate 10 models. Consequently, we got 10 independent duplicates for each modeling algorithm. AUC and overfitting values for 30 machine learning modeling algorithms were shown in table 2. Among 30 modeling algorithms, the minimum and maximum of AUC in set 2 were 0.557 (SD 0.051) and 0.866 (SD 0.082), respectively. The best algorithm was an ensemble one from five models that used oversampling for data balance after data imputing, and without data binning (table 3). Nine variables were used to build this model, which were age, gender, whether the prior fasting blood glucose was under control, duration of the current treatment regimen, diet adjustment or not, the daily cost of medications, fasting blood glucose value, hyperlipidemia and BMI after the backward variable selection process.

**Table 2:** AUC and overfitting values for 30 machine learning models

Imputing or not	Sampling methods	Binning or not	Screening methods	Model methods	Num of Variables	Num of Samples	AUC_TR	AUC_TE	AUC_Set 2	OF1	OF2
Not	Not	Not	Not	$D	16	167	0.693±0.025	0.621±0.054	0.737±0.062	1.125±0.122	0.849±0.111
Not	Not	Not	Forward and stepwise	$S	3	167	0.551±0.033	0.577±0.078	0.557±0.051	0.974±0.162	1.039±0.131
Not	Not	Not	Backward	$L	5	167	0.659±0.019	0.658±0.039	0.687±0.052	1.005±0.072	0.964±0.102
Not	Not	Yes	Forward and stepwise	$S	3	167	0.551±0.033	0.577±0.078	0.577±0.051	0.974±0.162	1.039±0.131
Not	Not	Yes	Backward	$S	3	167	0.551±0.033	0.577±0.078	0.577±0.051	0.974±0.162	1.039±0.131
Not	Undersampling	Not	Not	$XF	16	98	0.778±0.025	0.827±0.080	0.744±0.085	0.949±0.102	1.130±0.198
Not	Undersampling	Not	Forward and stepwise	$L	3	98	0.679±0.032	0.660±0.075	0.664±0.067	1.044±0.142	1.008±0.192
Not	Undersampling	Not	Backward	$L	3	98	0.679±0.032	0.660±0.075	0.664±0.067	1.044±0.142	1.008±0.192
Not	Undersampling	Yes	Forward and stepwise	$KNN	4	98	0.725±0.028	0.674±0.074	0.715±0.070	1.086±0.122	0.955±0.169
Not	Undersampling	Yes	Backward	$XF	5	98	0.755±0.047	0.753±0.074	0.725±0.058	1.013±0.136	1.044±0.125
Not	Oversampling	Not	Not	$R	16	263	0.781±0.021	0.770±0.040	0.758±0.070	1.017±0.060	1.028±0.150
Not	Oversampling	Not	Backward	$XF	5	263	0.814±0.031	0.799±0.037	0.761±0.094	1.021±0.073	1.066±0.155
Not	Oversampling	Not	Forward and stepwise	$XF	4	263	0.716±0.020	0.726±0.026	0.690±0.068	0.987±0.048	1.064±0.133
Not	Oversampling	Yes	Forward and stepwise	$XF	4	263	0.834±0.030	0.821±0.031	0.782±0.080	1.018±0.068	1.060±0.112
Not	Oversampling	Yes	Backward	$XF	7	263	0.864±0.028	0.856±0.022	0.813±0.127	1.010±0.040	1.088±0.261
Yes	Not	Not	Not	$D	16	315	0.725±0.012	0.678±0.047	0.703±0.051	1.074±0.087	0.973±0.131
Yes	Not	Yes	Forward and stepwise	$XF	5	315	0.812±0.024	0.760±0.048	0.757±0.056	1.073±0.089	1.008±0.091
Yes	Not	Not	Forward and stepwise	$XF	4	315	0.752±0.020	0.701±0.070	0.711±0.055	1.084±0.131	0.994±0.144
Yes	Not	Not	Backward	$XF	6	315	0.742±0.019	0.734±0.063	0.734±0.066	1.017±0.094	1.012±0.160
Yes	Not	Yes	Backward	$B	6	315	0.729±0.019	0.718±0.099	0.714±0.100	1.034±0.151	1.034±0.266
Yes	Undersampling	Not	Not	$B	16	199	0.785±0.032	0.811±0.087	0.778±0.063	0.980±0.126	1.052±0.170
Yes	Undersampling	Yes	Forward and stepwise	$XF	4	199	0.701±0.027	0.665±0.074	0.722±0.050	1.067±0.135	0.927±0.130
Yes	Undersampling	Not	Forward and stepwise	$S	4	199	0.685±0.022	0.658±0.069	0.702±0.053	1.053±0.137	0.946±0.146
Yes	Undersampling	Yes	Backward	$S	5	199	0.699±0.015	0.754±0.083	0.733±0.052	0.938±0.113	1.034±0.143
Yes	Undersampling	Not	Backward	$KNN	5	199	0.740±0.029	0.738±0.082	0.736±0.065	1.017±0.143	1.013±0.165
Yes	Oversampling	Not	Not	$XF	16	513	0.916±0.030	0.869±0.041	0.862±0.123	1.056±0.052	1.039±0.243
Yes	Oversampling	Yes	Forward and stepwise	$B	7	513	0.857±0.023	0.824±0.039	0.849±0.072	1.042±0.052	0.978±0.118
Yes	Oversampling	Not	Forward and stepwise	$XF	8	513	0.907±0.031	0.861±0.039	0.843±0.115	1.054±0.049	1.049±0.230
Yes	Oversampling	Not	Backward	$XF	9	513	0.907±0.024	0.871±0.030	**0.866±0.082**	1.041±0.036	1.017±0.134
Yes	Oversampling	Yes	Backward	$B	9	513	0.865±0.032	0.823±0.050	0.839±0.107	1.054±0.070	1.003±0.191

OF1 was calculated using the formula: AUC_Set 1 training set_ /AUC_Set 1 testing set_, and OF2, AUC_Set 1 testing set_ /AUC_Set 2_.

The bold value was the maximum AUC_Set 2_ of 30 algorithms.

AUC, area under the receiver operating characteristic curve; AUC_Set 2, AUC of set 2; AUC_TE, AUC of set 1 testing set; AUC_TR, AUC of set 1 training set; $B, Bayesian network; $D, discriminant model; $KNN, KNN algorithm; $L, logistic regression model; $R, CHAID; $S, SVM; $XF, the ensemble model.

**Table 3:** Assessment of models by different algorithms of the selected data governance

Models	AUC	Precision	Recall	F1 score (Online supplementary file 1)
Bayesian network	0.764±0.029	0.729±0.055	0.717±0.042	0.721±0.025
KNN	0.838±0.018	0.813±0.046	0.636±0.062	0.712±0.039
SVM	0.765±0.044	0.728±0.059	0.589±0.078	0.647±0.046
C&R Tree	0.755±0.030	0.739±0.026	0.669±0.066	0.700±0.033
CHAID	0.770±0.041	0.792±0.035	0.655±0.049	0.716±0.031
Ensemble	**0.866±0.082**	**0.824±0.043**	**0.732±0.061**	**0.773±0.032**
P value	p<0.0001	p<0.0001	p<0.0001	p<0.0001

The bold value indicate the performance parameters of the best algorithm.

AUC, area under the receiver operating characteristic curve.

10.1136/bmjdrc-2019-001055.supp1 Supplementary data

### Overfitting risk of the models

OF1 and OF2 were used to evaluate the overfitting risk of the model. The overall OF1 value was 1.028±0.113, and its difference from 1 was statistically significant (Student’s t-test, p<0.0001). The OF2 value was 1.015±0.165, and no significant difference was observed between OF2 and 1 (signed-rank test, p=0.6268). Despite the statistical differences between OF1 and 1, we believe that the overfitting risks of all models were negligible because their mean values were very close to 1, and their differences between 1 were Gaussian distributed and SD values were small (which means few variances).

### Impact of modeling methods on predictive performance

The impacts of algorithms on predictive performance are shown in table 4. Data imputing can significantly improve the AUC of set 2 (imputing, 0.770±0.096 vs not imputing, 0.694±0.106). Oversampling and undersampling will help get a better AUC (oversampling, 0.806±0.107 and undersampling, 0.718±0.069 vs not sampling, 0.671±0.097). As the number of variables and samples increases, the prediction performances of the models were significantly improved. In addition, different predictive powers were shown among various methods.

**Table 4:** The impact of modeling approaches on predictive indicators

Approaches	AUC_TR				AUC_TE				AUC_Set 2				OF1				OF2			
	Univariate analysis		Multivariate analysis*		Univariate analysis		Multivariate analysis		Univariate analysis		Multivariate analysis		Univariate analysis		Multivariate analysis		Univariate analysis		Multivariate analysis	
	P value	MMD/R	P value	SE	P value	MMD/R	P value	SE	P value	MMD/R	P value	SE	P value	MMD/R	P value	SE	P value	MMD/R	P value	SE
Imputing or not	**<0.0001**†	**0.0793**	**0.0019**	−**0.3020**	**<0.0001**†	**0.0605**	0.0813	−0.2082	**<0.0001**†	**0.0759**	0.1719	−0.1687	**0.0394**†	**0.0231**	0.6812	−0.0635	0.1084†	0.0203	0.6829	−0.0634
Sampling methods	**<0.0001**†	**0.1695**	**0.0141**	−**0.2757**	**<0.0001**†	**0.1620**	0.2980	−0.1436	**<0.0001**†	**0.1349**	0.0840	−0.2471	0.7884†	0.0145	0.3667	−0.1615	0.5764†	0.0440	0.6617	0.0786
Binning or not	0.6441†	0.0053	0.7135	−0.0149	0.7837†	0.0009	0.8834	−0.0073	0.8258†	0.0028	0.7578	0.0160	0.9188†	0.0066	0.9212	−0.0064	0.6672†	0.0036	0.7668	−0.0193
Screening methods	**0.0119**†	**0.0489**	**0.0338**	**0.1102**	**0.0042**†	**0.0541**	**0.0024**	**0.1950**	**0.0091**‡	**0.0513**	**0.0352**	**0.1394**	0.2277‡	0.0242	0.1343	−0.1242	0.6512†	0.0213	0.4484	0.0631
Model methods	**<0.0001**†	**0.2015**	**<0.0001**	**0.2734**	**<0.0001**†	**0.1654**	**<0.0001**	**0.2864**	**<0.0001**†	**0.1739**	**<0.0001**	**0.2025**	**0.0143**†	**0.1166**	0.5617	−0.0386	**0.0271**†	**0.1274**	0.1616	0.0936
Num of Variables	**<0.0001**§	**0.6121**	**<0.0001**	**0.2905**	**<0.0001**§	**0.5197**	**<0.0001**	**0.3360**	**<0.0001**§	**0.5147**	**<0.0001**	**0.3134**	0.2593§	0.0653	0.4035	−0.0739	0.2219§	−0.0707	0.7425	0.0292
Num of Samples¶	**<0.0001**§	**0.6716**	**<0.0001**	**0.9020**	**<0.0001**§	**0.5083**	**0.0022**	**0.5584**	**<0.0001**§	**0.4949**	**<0.0001**	**0.3655**	0.0722§	0.1039	0.1972	0.3024	0.5946§	−0.0308	0.8326	−0.0497

Oversampling leading to abnormal distribution in all five indexes.

The bold values indicate the parameters of approaches which would significantly affect predictive indicators.

*Multiple linear regression was used for multivariate analysis.

†Kruskal-Wallis test.

‡General linear models for analysis of variance (ANOVA).

§Spearman correlation analysis.

¶The variance inflation factor (VIF) of variable ‘Num of Samples’ in multiregression model is 16.4146 (which is greater than 10), indicates multicollinearity that maybe exists and may make the model unstable; this variable may be severely collinear with imputing, binning, and sampling, so the multiple linear regression (MLR) model was re-established after the three variables were eliminated.

AUC, area under the receiver operating characteristic curve; AUC_Set 2, AUC of set 2; AUC_TE, AUC of set 1 testing set; AUC_TR, AUC of set 1 training set; MMD, maximum mean difference among levels; R, correlation coefficient; SE, standardized estimate.

Multilinear regression analysis showed that variable screening methods, algorithms, number of variables and sample size will affect the AUC of set 2 remarkably. The factor ‘sample size’ had the strongest impact on AUC of set 2 according to SE value (see table 3).

### Sample size assessment

As the size of sample data incorporated into the model from small to large, the AUC values of the testing sets continued to increase. When the sample size is extremely small (less than or equal to 30%), the SDs of AUC are large, and the values show significant discrete distribution trends. As the sample size increases, the above situation is alleviated.

## Discussion

The development of patient’s medication adherence predictive models facilitates individualized diabetes education and care.^19 20^ In our research, 30 machine learning-based modeling algorithms were developed for adherence prediction, which at most 16 variables were used to build the model. After examining data filling, sampling, binning, and variable screening methods, the best algorithm, whose mean AUC was 0.866±0.082, was selected. The model was validated by sensitivity, specificity, and overfitting risk, and excellent predictive capability was shown to identify non-adherence patients.

Although studies related to medication adherence predictive models are found in tuberculosis^14^ and heart failure,^15^ few studies have been retrieved that established and examined the predictive models in patients with type 2 diabetes mellitus (T2DM), for whom medication adherence is a key factor to treatment outcomes.^5 21 22^ Kumamaru H *et al*^23^ established a logistic regression model for the prediction of future drug compliance. In their study, the patient’s current drug dependence characteristic played the role of the main predictor. Data from 90 000 patients were analyzed in the study. However, the AUC of their model was 0.666 (95% CI 0.661 to 0.671), which was smaller than ours (0.866±0.082). That may be because they focused on patients newly initiating statins, used retrospective data and evaluated fewer variables while we focused on a smaller population (patients with T2DM), conducted a prospective study and examined more targeted variables.

The proportion of patients with good adherence in this study is 78.8%, which is comparable to data from Ethiopia^24^ (70.5%), the USA^25^ (70.0%) and white patients who were newly prescribed diabetes medications^26^ (62.5%). A systematic review showed that the rate of adherence to oral hypoglycemic agents ranged from 36%–93% and 62%–64% to insulin in patients with T2DM.^27^ Twenty-seven percent of Palestine refugees who have diabetes living in Jordan were not adherent to their regimens.^28^ Many variables are reportedly considered to be related to patient medication compliance such as gender, dyslipidemia, medication, and so on.^29 30^ To our knowledge, this study is the first to examine the factors and develop predictive models based on the factors such as the duration of the current medication regimen, whether the last fasting blood glucose was under control, and whether a reasonable diet plan had been implemented.

Data filling is an important way to solve data loss problems that frequently appear in real-world studies. Every record needs a value for each variable during the multivariate analysis process in spite of missing data are inevitable in real-world data sets. Deleting the entire patient’s record due to small parts of data missing will result in a large amount of information loss. It could be necessary to delete the variables and records under severe missing circumstances (eg, more than 70% of data missing) to reduce noise in the whole data. However, there is still no consensus about whether the absence of the remaining data should be filled or how to perform effective data padding. Several studies have reported interpolation methods under the situation of data missing.^31–33^ Specially, multiple imputation (MI) methods are a wonderful approach to handle data missing issues for the consideration of the imputation uncertainty.^34^ MI generates *m* data sets, obtains results from each data set and combines *m* results at the last step. In this study, 300 models were built by using one data set and one algorithm was selected for use. We have not figured out how to combine *m*×300 models and find one algorithm out. So we used simple imputation here. The results of our study showed that the predictive abilities of moles were significantly improved (p<0.0001) after data filling. Univariate and multivariate analyses also showed that the increase in the available sample size would significantly enhance the models’ predictive performances which maybe also partly due to data imputation.

Undersampling and oversampling are the main methods used to solve the issues that result from sample imbalance which also frequently happen in medical-related research data,^35^ such as the occurrence of adverse reactions and non-adverse reactions.^36^ Undersampling reduces the number of samples at the level of more samples while oversampling increases the number of samples at the level of fewer samples. Overestimation of AUC may occur on account of oversampling because of the existence of a large number of duplicate samples and some records are used to test the model after being used to train the model. Therefore, in this study, the data partitioning process was designed twice to produce a non-repeating set of verification samples, which is set 2, in order to ensure the credibility of the results. In addition, this study shows that oversampling is a key measure to improve predictive sensitivity and specificity (p<0.0001).

### Strengths and limitations

There are several innovations in this study. First, we are more focused on collecting and evaluating variables that may be relevant to medication compliance in the real-world environment, which may increase the prediction accuracy of the model based on a limited sample size. Second, the model with the best prediction effect was found based on as many as 300 models built according to a variety of data processing methods and ten times randomly partitioning of data. Finally, we have innovatively tried a sample size posterior verification method based on the relative relationship between sample size and AUC, which can provide a reference for sample size analysis of predictive studies. However, limitations also exist in this study. First, in the sample size verification results, there is no inflection point in the AUC curve as the sample size increases, indicating that there is still a need for a further increase in the sample size. The second one is that we need to work with more centers to collect more data to optimize the model and verify the applicability of the model in healthcare facilities. Lastly, although we classified the patients’ last test results to reduce accuracy, for some variables, recall bias still exists.

## Conclusion

In Sichuan Province, the southwest region of China, 21.20% of patients with T2D were not adherent to their medication regimens. The duration of the current medication regimen, former blood glucose level, and eating habits can be key factors in predicting patient medication compliance. By performing data padding when the data have a small number of missing values, balanced sampling in the imbalanced data, and model training using a variety of algorithms, the better predictive models will be obtained. After establishing and evaluating a large number of models, we got a predictive model with potential clinical value for preventing non-adherence risks of patients with T2D.

10.1136/bmjdrc-2019-001055.supp2 Supplementary data

## References

[1] Ogurtsova K, da Rocha Fernandes JD, Huang Y, et al IDF diabetes atlas: global estimates for the prevalence of diabetes for 2015 and 2040. Diabetes Res Clin Pract 2017;128:40–50. 10.1016/j.diabres.2017.03.024 28437734

[2] Bertoni AG, Krop JS, Anderson GF, et al Diabetes-Related morbidity and mortality in a national sample of U.S. elders. Diabetes Care 2002;25:471–5. 10.2337/diacare.25.3.471 11874932

[3] Jacobson AM Impact of improved glycemic control on quality of life in patients with diabetes. Endocr Pract 2004;10:502–8. 10.4158/EP.10.6.502 16033724

[4] Stolar M Glycemic control and complications in type 2 diabetes mellitus. Am J Med 2010;123:S3–11. 10.1016/j.amjmed.2009.12.004 20206730

[5] Gordon J, McEwan P, Idris I, et al Treatment choice, medication adherence and glycemic efficacy in people with type 2 diabetes: a UK clinical practice database study. BMJ Open Diabetes Res Care 2018;6:e000512 10.1136/bmjdrc-2018-000512 PMC5942418 29755756

[6] Lin L-K, Sun Y, Heng BH, et al Medication adherence and glycemic control among newly diagnosed diabetes patients. BMJ Open Diabetes Res Care 2017;5:e000429 10.1136/bmjdrc-2017-000429 PMC5574459 28878942

[7] Zhou FL, Yeaw J, Karkare SU, et al Impact of a structured patient support program on adherence and persistence in basal insulin therapy for type 2 diabetes. BMJ Open Diabetes Res Care 2018;6:e000593 10.1136/bmjdrc-2018-000593 PMC6307592 30622720

[8] Wolever RQ, Dreusicke MH Integrative health coaching: a behavior skills approach that improves HbA1c and pharmacy claims-derived medication adherence. BMJ Open Diabetes Res Care 2016;4:e000201 10.1136/bmjdrc-2016-000201 PMC4873948 27239318

[9] Swarna Nantha Y, Haque S, Swarna Nantha H The development of an integrated behavioural model of patient compliance with diabetes medication: a mixed-method study protocol. Fam Pract 2019;36:581–6. 10.1093/fampra/cmy119 30534941 PMC6781935

[10] Capoccia K, Odegard PS, Letassy N Medication adherence with diabetes medication: a systematic review of the literature. Diabetes Educ 2016;42:34–71. 10.1177/0145721715619038 26637240

[11] Pattnaik S, Ausvi SM, Salgar A, et al Treatment compliance among previously diagnosed type 2 diabetics in a rural area in southern India. J Family Med Prim Care 2019;8:919 10.4103/jfmpc.jfmpc_23_19 31041225 PMC6482764

[12] Lo-Ciganic W-H, Donohue JM, Thorpe JM, et al Using machine learning to examine medication adherence thresholds and risk of hospitalization. Med Care 2015;53:720–8. 10.1097/MLR.0000000000000394 26147866 PMC4503478

[13] Taranik M, Kopanitsa G Using machine learning for personalized patient adherence level determination. Stud Health Technol Inform 2019;261:174–8. 31156111

[14] Killian JA, Wilder B, Sharma A, et al Learning to prescribe interventions for tuberculosis patients using digital adherence data, 2019.

[15] Karanasiou GS, Tripoliti EE, Papadopoulos TG, et al Predicting adherence of patients with HF through machine learning techniques. Healthc Technol Lett 2016;3:165–70. 10.1049/htl.2016.0041 27733922 PMC5048333

[16] Chowdhury R, Khan H, Heydon E, et al Adherence to cardiovascular therapy: a meta-analysis of prevalence and clinical consequences. Eur Heart J 2013;34:2940–8. 10.1093/eurheartj/eht295 23907142

[17] Cramer JA, Benedict A, Muszbek N, et al The significance of compliance and persistence in the treatment of diabetes, hypertension and dyslipidaemia: a review. Int J Clin Pract 2008;62:76–87. 10.1111/j.1742-1241.2007.01630.x 17983433 PMC2228386

[18] Zhang Z Missing data imputation: focusing on single imputation. Ann Transl Med 2016;4:9 10.3978/j.issn.2305-5839.2015.12.38 26855945 PMC4716933

[19] Eggerth A, Hayn D, Schreier G Medication management needs information and communications technology-based approaches, including telehealth and artificial intelligence. Br J Clin Pharmacol 2019. doi:10.1111/bcp.14045. [Epub ahead of print: 04 Jul 2019]. PMC7495302 31271668

[20] Riegel B, Knafl GJ Electronically monitored medication adherence predicts hospitalization in heart failure patients. Patient Prefer Adherence 2013;8:1–13. 10.2147/PPA.S54520 24353407 PMC3862652

[21] Boye KS, Curtis SE, Lage MJ, et al Associations between adherence and outcomes among older, type 2 diabetes patients: evidence from a Medicare supplemental database. Patient Prefer Adherence 2016;10:1573–81. 10.2147/PPA.S107543 27574406 PMC4993402

[22] Huber CA, Rapold R, Brüngger B, et al One-Year adherence to oral antihyperglycemic medication and risk prediction of patient outcomes for adults with diabetes mellitus: an observational study. Medicine 2016;95:e3994 10.1097/MD.0000000000003994 27368004 PMC4937918

[23] Kumamaru H, Lee MP, Choudhry NK, et al Using previous medication adherence to predict future adherence. J Manag Care Spec Pharm 2018;24:1146–55. 10.18553/jmcp.2018.24.11.1146 30362915 PMC10397923

[24] Ayele AA, Tegegn HG, Ayele TA, et al Medication regimen complexity and its impact on medication adherence and glycemic control among patients with type 2 diabetes mellitus in an Ethiopian General Hospital. BMJ Open Diabetes Res Care 2019;7:e000685 10.1136/bmjdrc-2019-000685 PMC6606061 31321061

[25] Ratanawongsa N, Karter AJ, Parker MM, et al Communication and medication refill adherence: the diabetes study of northern California. JAMA Intern Med 2013;173:210–8. 10.1001/jamainternmed.2013.1216 23277199 PMC3609434

[26] Fernández A, Quan J, Moffet H, et al Adherence to newly prescribed diabetes medications among insured Latino and white patients with diabetes. JAMA Intern Med 2017;177:371–9. 10.1001/jamainternmed.2016.8653 28114642 PMC5814298

[27] Cramer JA A systematic review of adherence with medications for diabetes. Diabetes Care 2004;27:1218–24. 10.2337/diacare.27.5.1218 15111553

[28] Canali G, Tittle V, Seita A Medication adherence by Palestine refugees living in Jordan who have diabetes: a cross-sectional study. Lancet 2018;391:S13 10.1016/S0140-6736(18)30379-9 29553410

[29] Jaam M, Awaisu A, Ibrahim MI, et al Synthesizing and appraising the quality of the evidence on factors associated with medication adherence in diabetes: a systematic review of systematic reviews. Value Health Reg Issues 2017;13:82–91. 10.1016/j.vhri.2017.09.001 29073997

[30] McGovern A, Tippu Z, Hinton W, et al Comparison of medication adherence and persistence in type 2 diabetes: a systematic review and meta-analysis. Diabetes Obes Metab 2018;20:1040–3. 10.1111/dom.13160 29135080

[31] Gan X, Liew AW-C, Yan H Microarray missing data imputation based on a set theoretic framework and biological knowledge. Nucleic Acids Res 2006;34:1608–19. 10.1093/nar/gkl047 16549873 PMC1409680

[32] Ozkan H, Pelvan OS, Kozat SS Data imputation through the identification of local anomalies. IEEE Trans Neural Netw Learn Syst 2015;26:2381–95. 10.1109/TNNLS.2014.2382606 25608311

[33] Zhang Z, Ho KM, Hong Y Machine learning for the prediction of volume responsiveness in patients with oliguric acute kidney injury in critical care. Crit Care 2019;23:112 10.1186/s13054-019-2411-z 30961662 PMC6454725

[34] Zhang Z Multiple imputation for time series data with Amelia package. Ann Transl Med 2016;4:56 10.3978/j.issn.2305-5839.2015.12.60 26904578 PMC4740012

[35] Koutsiana E, Chytas A, Vaporidi K, et al Smart alarms towards optimizing patient ventilation in intensive care: the driving pressure case. Physiol Meas 2019;40:095006 10.1088/1361-6579/ab4119 31480025

[36] Dai H-J, Wang C-K Classifying adverse drug reactions from imbalanced Twitter data. Int J Med Inform 2019;129:122–32. 10.1016/j.ijmedinf.2019.05.017 31445246

