# Supplementary figures and images for: Predictive models of medication non-adherence risks of patients with T2D based on multiple machine learning algorithms
Source: BMJ Open Diabetes Res Care. 2020 Mar 9;8(1):e001055. doi: 10.1136/bmjdrc-2019-001055 (PMC7064141; doi:10.1136/bmjdrc-2019-001055)

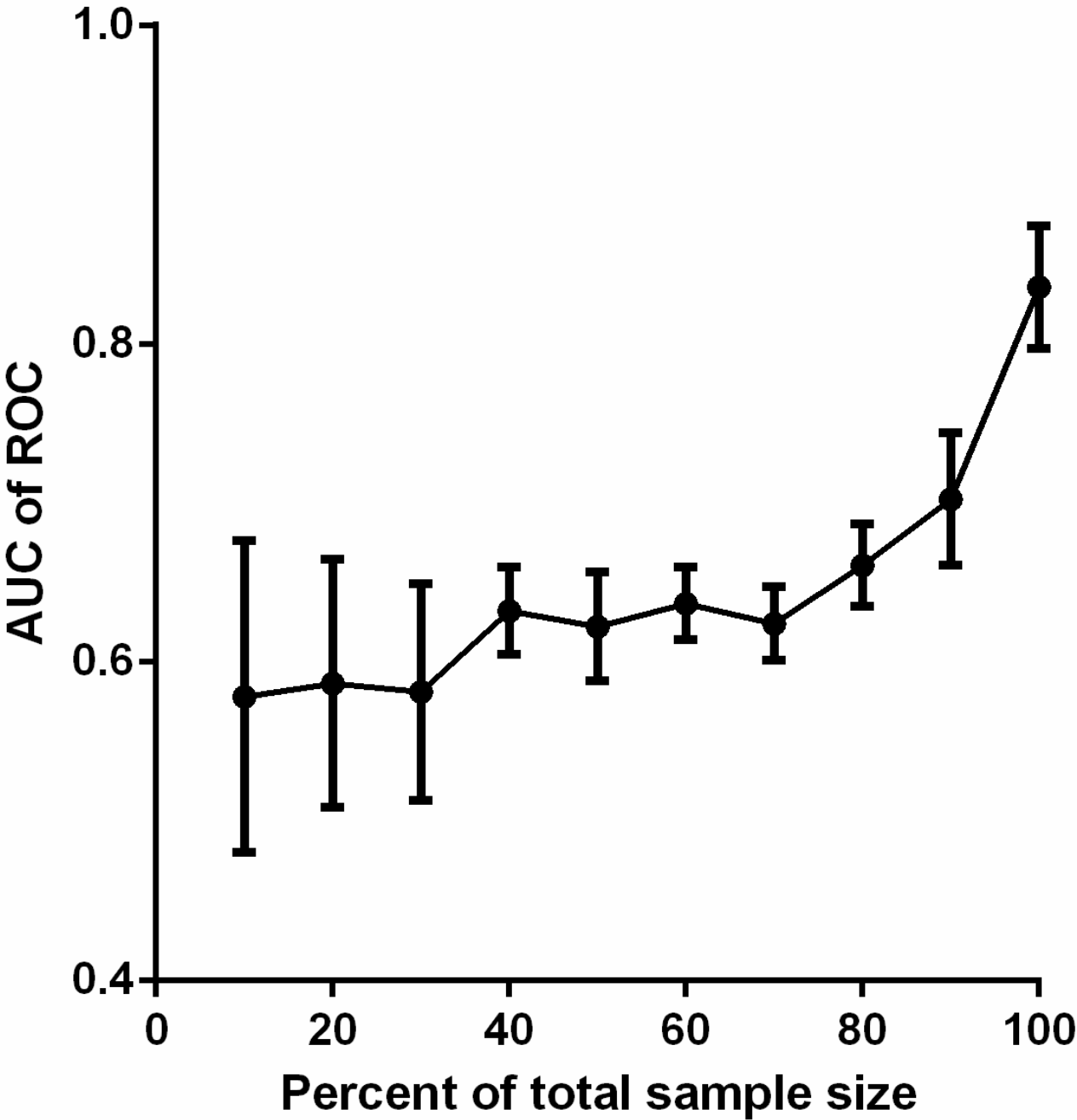

Supplement: Supplementary data [file bmjdrc-2019-001055supp002.pdf]
